# Clinical characteristics and outcomes of primary sclerosing cholangitis and ulcerative colitis in Japanese patients

**DOI:** 10.1371/journal.pone.0209352

**Published:** 2018-12-20

**Authors:** Junichiro Kumagai, Takashi Taida, Sadahisa Ogasawara, Tomoo Nakagawa, Yotaro Iino, Ayako Shingyoji, Kentaro Ishikawa, Naoki Akizue, Mutsumi Yamato, Koji Takahashi, Yuki Ohta, Shinsaku Hamanaka, Kenichiro Okimoto, Masato Nakamura, Hiroshi Ohyama, Keiko Saito, Yuko Kusakabe, Daisuke Maruoka, Shin Yasui, Tomoaki Matsumura, Harutoshi Sugiyama, Yuji Sakai, Rintaro Mikata, Makoto Arai, Tatsuro Katsuno, Toshio Tsuyuguchi, Naoya Kato

**Affiliations:** Department of Gastroenterology, Graduate School of Medicine, Chiba University, Chiba, Japan; Laval University, CANADA

## Abstract

**Background:**

In Western countries, most patients with primary sclerosing cholangitis (PSC) have concurrent ulcerative colitis (UC). The number of patients with UC in East Asia has increased markedly over the past two decades. However, current clinical features of PSC and of PSC associated with UC (PSC-UC) have not yet been clarified in East Asia, particularly in Japan. We aimed to reveal the clinical courses and associations with UC in Japanese patients with PSC from the mutual viewpoint of PSC and UC.

**Methods:**

We retrospectively retrieved medical records of patients with PSC (69) and UC (1242) who were diagnosed at Chiba University Hospital between June 1991 and August 2017.

**Results:**

In the present cohort, 37 patients had PSC-UC; the cumulative risks of PSC in patients with UC and of UC in patients with PSC were 3.0% and 53.6%, respectively. We confirmed similar distinctive results by a Japanese nationwide survey, noting that younger patients with PSC had a notably high possibility of association with UC. From the viewpoint of the UC cohort, the occurrence of right-sided disease was significantly higher in patients with PSC-UC than in those with UC (16.2% vs. 4.2%, P = 0.003). Pancolitis was more commonly observed in PSC-UC, and proctits/left-sided colitis was less commonly found in patients with UC. The number of patients with young-onset PSC-UC may be increasing similar to an increase in patients with UC in Japan.

**Conclusions:**

In our cohort, the comorbidity rate of PSC-UC was higher than that obtained in previous reports. The incidence of PSC-UC and UC may increase in the future in East Asia, particularly in Japan.

## Introduction

Primary sclerosing cholangitis (PSC) is a chronic disease characterized by chronic inflammation and fibrotic obliteration of the intra- and/or extrahepatic bile duct, resulting in bile stasis and hepatic fibrosis [1]. There is no effective medical treatment for this condition and liver transplantation represents the treatment choice in patients with end-stage liver disease [1]. PSC is associated commonly with underlying inflammatory bowel disease (IBD), mainly ulcerative colitis (UC) [1]. In Western countries, approximately 52% to 90% of patients with PSC have concurrent UC [2–8]. Several previous systemic reviews have shown that incidence and prevalence vary geographically [9,10]. In north Europe, the incidence and prevalence have reached 1.22 and 16.2 per 100,000 people, respectively [11]. In addition, the numbers and incidence rate of PSC have increased temporally worldwide. Meanwhile, the number of patients with UC, as well as with other IBDs, continues to increase in Western countries [12,13]. Several current reports suggest that the numbers of patients with PSC associated with UC (PSC-UC) have increased gradually in some European populations [8,14].

Although there have been few population-based studies from Asia, the incidence and prevalence rates of PSC in Asian patients are believed to be lower than those in Western countries. Takikawa and Manabe [15] and Tanaka et al. [16] reported the characteristics of PSC in Japanese patients and found that age distribution has two peaks, and that the younger age group had a high frequency of the disease being associated with IBD, especially UC. The comorbidity rates of IBD and UC in patients with PSC in the current Japanese population were 40% and 32%, respectively, which were lower than those of Western cohorts [17].

Currently, the prevalence of UC has increased rapidly in East Asian countries [12,18–20]. Nowadays, Japan is the second country with the highest number of UC patients in the world, after the United States [18–21]. Global spread of UC, especially in East Asia, appears to be associated with Westernization of diets and environments, which affects the intestinal microbiome and increases the risk of UC in genetically susceptible individuals [12,22]. Although it remains controversial in detail, PSC and UC are intractable and considered to involve an immune mechanism [23]. Since conditions in Japanese patients with UC are changing rapidly, research analyzing clinical features in Japanese patients with PSC, which especially focuses on PSC-UC, is essential. We assessed the characteristics of Japanese patients with PSC from the mutual viewpoint of PSC and UC.

## Patients and methods

### Patients

Our study was approved by the Research Ethics Committee of the Graduate School of Medicine, Chiba University (approval number 2603, 2898). We had access to information that could identify individual patients during or after data collection. Patient data were made anonymous and de-identified before analysis.

### Study population and case ascertainment

Chiba University Hospital is the only university hospital and the single high-volume center (with more than 800 beds, and established gastroenterology and hepatology, digestive tract surgery, and hepato-biliary surgery departments) in Chiba City, which is inhabited by nearly 1 million people (the 12th largest population in Japan). Moreover, many patients are referred from community hospitals in Chiba City or from hospitals outside of Chiba City to the university hospital for advanced diagnostic and/or therapeutic procedures.

During the study period of June 1991 to August 2017, patients with PSC and UC were identified from registers in the electronic health records of Chiba University Hospital, which included in- and outpatients of all departments at our hospital. We used the keywords ‘PSC’ or ‘PSC suspected’ to identify PSC patients, while ‘IBD’ or ‘IBD suspected’ was for UC patients. We extracted PSC and UC patients, including uncertain cases, and diagnostics were confirmed by two medical specialists for PSC (Kumagai J and Tsuyuguchi T) and UC (Taida T and Nakagawa T), respectively.

Medical records, including clinical, pathological, endoscopy, and radiological records, were retrieved in all cases retrospectively and were validated according to the standard protocol as described below. Based on the protocol, we collected data on gender, date of birth, date of diagnosis, date of death, data lost to follow-up, history of smoking and alcohol abuse, records of treatments for PSC and UC, and occurrence of hepatobiliary and colorectal carcinoma.

PSC was diagnosed according to the conventional radiographic criteria [3]. All patients were examined by endoscopic retrograde cholangiography (ERCP) and/or magnetic resonance cholangiopancreatography (MRCP). Liver biopsy was done if needed. Small duct PSC was defined as disease with normal cholangiography but with features of PSC on histopathology from liver biopsy and positive clinical features [24]. Thus, in case of small duct PSC, we diagnosed PSC based on hepatic histological findings [25]. The confluence of the bile duct at the hilum was used to distinguish between intra- and extrahepatic biliary systems. Overlap autoimmune hepatitis (AIH) was diagnosed based on published simplified criteria for autoimmune hepatitis with a cut-off score of 6 [26].

During the follow-up period, we identified the time point when patients first became liver transplant candidacies and were first recognized to have a dominant stricture (DS). In our study, we defined liver transplantation candidacies as those with the following characters: (1) Child-Pugh classification C, (2) recurrent cholangitis at least once per month, (3) uncontrollable ascites, (4) uncontrollable itchiness, or (5) history of esophageal gastric varix rupture [2,3,27]. The criteria of DS in our study were defined as follows: (1) strictures of the common bile duct measuring <1.5 mm in diameter, and (2) strictures of the common hepatic duct with a diameter <1.0 mm within 2 cm from the bifurcation at the hilum [28].

We reviewed records of colonoscopy and histological findings and diagnosed UC and other IBDs according to the latest guidelines [29]. Based on the previous study [30], the extent of UC was classified by the records of colonoscopy as follows: proctitis type (affected rectum only), left-sided type (mainly affected middle transverse to rectum), right-sided type (mainly affected cecum to middle transverse), and pancolitis type (affected entire large intestine). In our study, we defined UC flare as the presence of symptoms requiring hospitalization or requiring a change in medical treatment except 5-aminosalicylic acid (5-ASA) [31,32].

### Statistical analysis

The Pearson χ2 test or Fisher exact test was used to compare demographic and clinical characteristics as appropriate. The Mann Whitney U test was used to analyze continuous data with non-normal distribution. The Kaplan-Meier method was performed to estimate duration of survival, liver transplantation free survival, and time to liver transplantation candidacy, and the log-rank test determined statistical significance. Duration of survival was defined as the time from the day of diagnosis until death, duration of liver transplantation free survival as the time from the day of diagnosis until either death or liver transplantation, and duration of onset of PSC to liver transplantation candidacy as the time from the day of onset until the day the patient was deemed a liver transplantation candidacy. Censoring date was defined as the date of last follow-up. A P value of <0.05 was considered statistically significant. All statistical analyses were performed using SPSS statistical software (version 25; SPSS-IBM, Chicago, IL, USA).

## Results

### Population of our study

**Fig 1:**
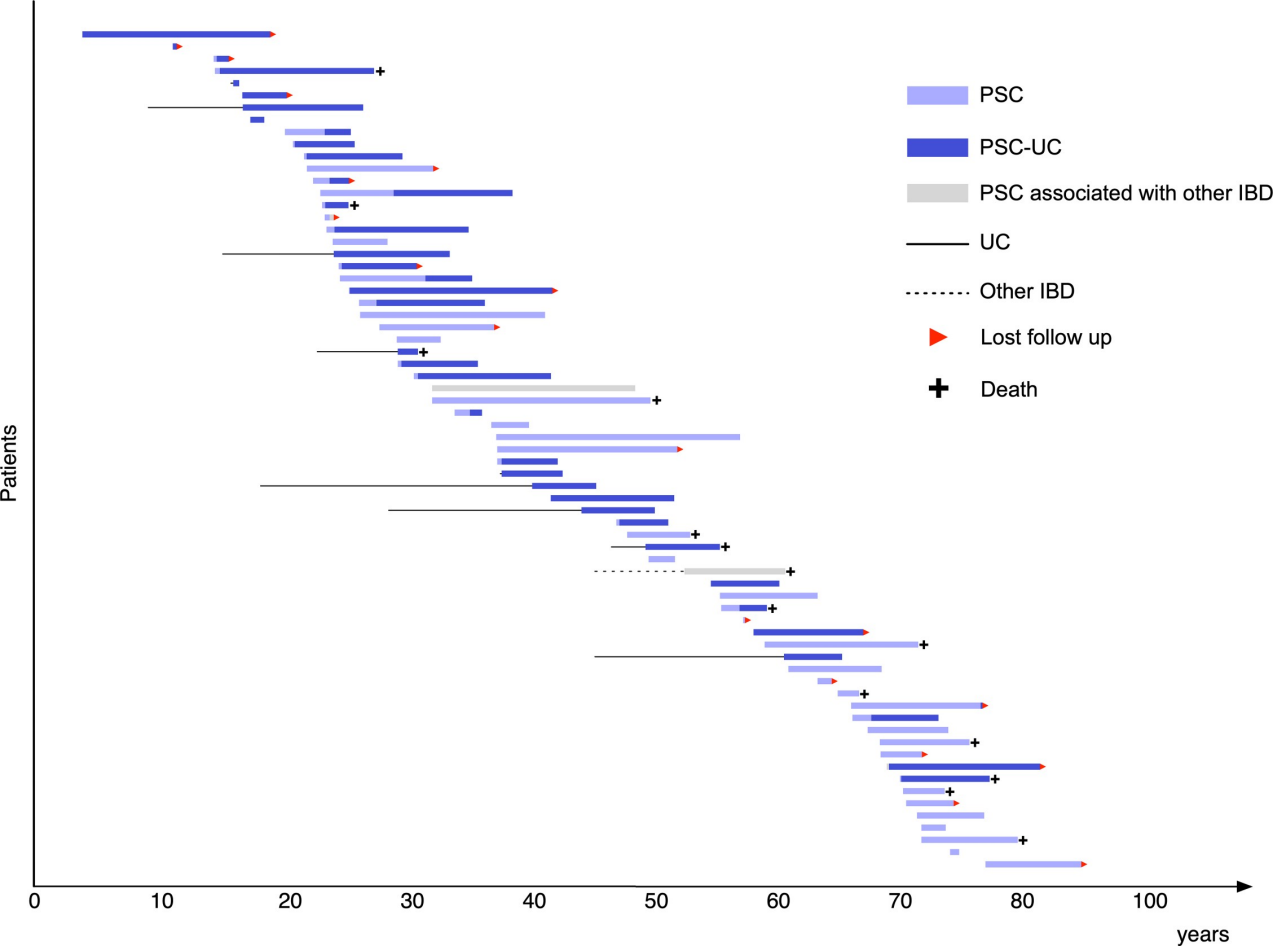
Clinical courses of 69 patients with PSC focusing on age at onset and disease duration.

We retrieved medical records of 200 and 2589 patients with suspected PSC and IBD, respectively, and identified 69 with PSC and 1242 with UC. During follow-up, PSC-UC was found in 37 patients: the cumulative risks of PSC in patients with UC and of UC in patients with PSC were 3.0% and 53.6%, respectively. Synchronous other IBDs with PSC were present in three patients (4.3%), including two (2.9%) with Crohn’s disease and one (1.4%) with IBD unclassified. Fig 1 demonstrates the clinical courses of 69 patients with PSC. Median follow-ups in the PSC and UC cohorts were 6.1 and 7.7 years, respectively.

### Analysis from the viewpoint of PSC population

Patient characteristics and outcomes comparing all those with PSC, PSC-UC, and PSC without UC are shown in Table 1. Most patients were male (62.3%), nonsmokers (82.6%), and had intra- and extrahepatic involvement (89.9%). Median ages of patients with PSC-UC and PSC without UC were 26 and 57 years, respectively (P < 0.001). Two peaks of age distribution at PSC diagnosis are indicated in Fig 1 as well. Patients aged 20–30 years and 60 years were at high risk of PSC. Median duration from diagnosis of UC to diagnosis of PSC was 0.2 years (range: −10.6–22.2), and 23 of 37 (62.1%) patients with PSC-UC were diagnosed with PSC and UC within a year, and 7 of 37 (18.9%) PSC-UC patients were diagnosed with UC > 1 year after PSC diagnosis. There were no significant differences in patient characteristics and outcomes between the PSC-UC and PSC without UC cohorts except for age at diagnosis of PSC. Only 3 of 23 (13.0%) patients with liver transplantation candidacy undergo liver transplantation, and the outcomes for overall survival and liver transplant-free survival were similar. However, patients with PSC without UC were more likely to become liver transplant candidacies than those with PSC-UC (P = 0.089).

**Table 1:** Patients’ characteristics and outcomes of PSC.

Demographics/Characteristics	PSC (*n* = 69)	PSC-UC (*n* = 37)	PSC without UC(*n* = 32)	*p*
**Sex**, male, *n* (%)	43 (62.3)	26 (70.3)	17 (53.1)	0.143^a^
**Median age at PSC diagnosis(range)**	38 (4–78)	26 (4–71)	57 (17–78)	< 0.001^b^
**Extent of PSC**, *n* (%)				
Intrahepatic involvement	5 (7.2)	3 (8.1)	2 (6.3)	1.000 ^a^
Extrahepatic involvement	1 (1.4)	0 (0.0)	1 (3.1)	0.464 ^a^
Both intra- and extrahepatic involvement	62 (89.9)	33 (89.2)	29 (90.6)	1.000 ^a^
**Small duct PSC,** *n* (%)	1 (1.4)	1 (2.7)	0 (0.0)	1.000^a^
**AIH overlap,** *n* (%)	2 (2.9)	1 (2.7)	1 (3.1)	1.000 ^a^
**Liver biopsy,** *n* (%)	23 (33.3)	12 (32.4)	11 (34.4)	0.865 ^a^
**Nonsmoker,** *n* (%)	57 (82.6)	31 (83.8)	26 (81.2)	1.000 ^a^
**Alcohol abuse,** *n* (%)	3 (4.3)	2 (5.4)	1 (3.1)	1.000 ^a^
**Medications,** *n* (%)				
UDCA	62 (89.9)	31 (83.8)	31 (96.8)	0.113 ^a^
Bezafibrate	25 (36.2)	12 (32.4)	13 (40.6)	0.480 ^a^
Inchin ko to	21 (30.4)	12 (32.4)	9 (28.1)	0.900 ^a^
**Liver transplantation candidacy**, *n* (%)	23 (33.3)	9 (24.3)	14 (43.8)	0.089^a^
**Undergoing liver transplantation**, *n* (%)	3 (4.4)	2 (5.4)	1 (3.1)	1.000 ^a^
**Colorectal cancer,** *n* (%)	3 (4.4)	2 (5.4)	1 (3.1)	1.000 ^a^
**Cholangiocarcinoma,** *n* (%)	11 (15.9)	4 (10.8)	7 (21.8)	0.356 ^a^
**Median duration from diagnosis of PSC to cholangiocarcinoma, years (range)**	1.10 (0.02–16.9)	2.08 (0.41–3.14)	1.03 (0.02–16.9)	0.365 ^c^
**Median survival from diagnosis of PSC, years (95% CI)**	18.0 (11.0–25.0)	Undefined	18.0 (7.7–28.3)	0.303^c^
**5-year survival rate from diagnosis of PSC (%)**	91.3	93.5	89.0	
**10-year survival rate from diagnosis of PSC (%)**	74.3	82.7	64.7	
**Median liver transplantation free survival from diagnosis of PSC, years (95% CI)**	18.0 (10.2–25.8)	Undefined	18.0 (7.7–28.3)	0.244 ^c^
**5-year liver transplantation free survival rate from diagnosis of PSC (%)**	89.7	90.3	89.0	
**10-year liver transplantation free survival rate from diagnosis of PSC (%)**	72.4	79.2	64.7	
**Median duration from diagnosis of PSC to the liver transplantation candidacy, years (95% CI)**	16.9 (7.9–25.9)	Undefined	10.8 (5.1–16.4)	0.218 ^c^
**5-year liver transplantation candidacy rate from diagnosis of PSC (%)**	26.9	25.6	28.7	
**10-years liver transplantation candidacy rate from diagnosis of PSC (%)**	41.4	32.4	49.7	

Abbreviations: PSC, primary sclerosing cholangitis; UC, ulcerative colitis; 95% CI, 95% Confidence interval.

^a^ p values were calculated using the Pearson χ2 test or Fisher exact test.

^b^ p values were calculated using the Mann Whitney U test.

^c^ p values were calculated using the log-rank test.

### Clinical courses of Japanese PSC patients

**Fig 2:**
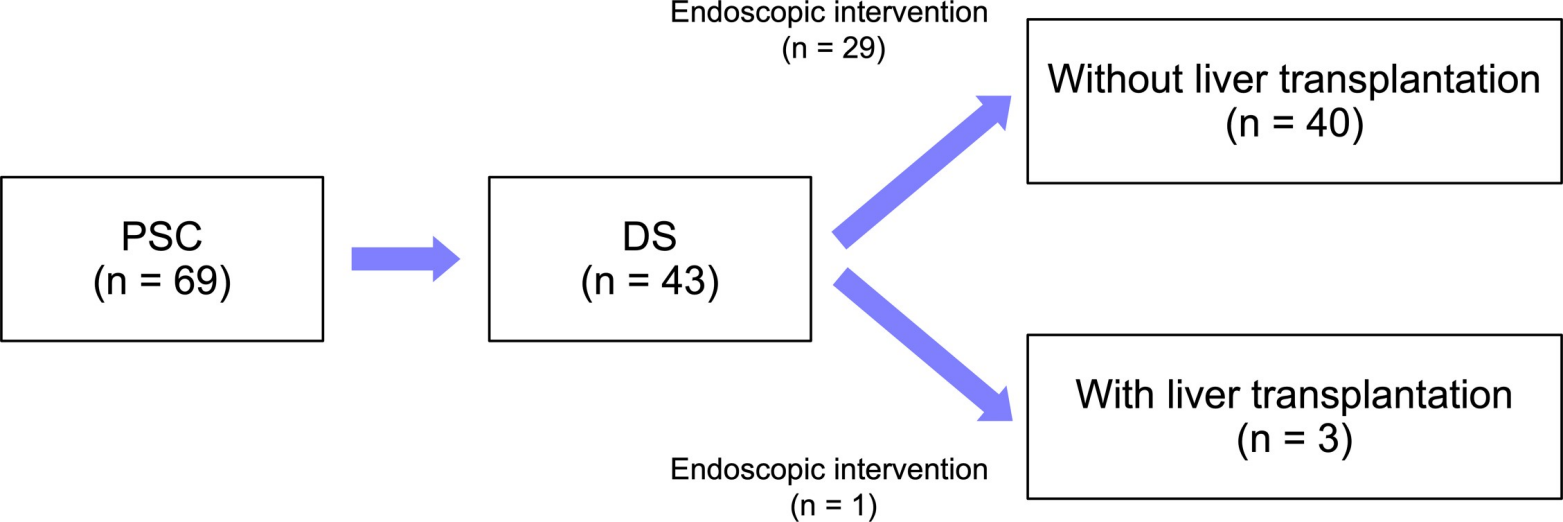
Progression from initial diagnosis to DS in 69 patients with PSC. DS, dominant stricture; PSC, primary sclerosing cholangitis.

Most patients were administered ursodeoxycholic acid (UDCA; 89.9%), followed by bezafibrate (36.2%), and Inchin ko-to (herbal medicine, TJ-135; 30.4%) [33,34]. We identified 43 patients (62.3%) with DS and 23 (33.3%) who were liver transplantation candidacies (Fig 2). Median durations from onset of PSC to DS and liver transplantation candidacy were 4.3 (95% CI, 1.4–7.2) and 16.9 (95% CI, 7.9–25.9) years, respectively. In our cohorts, only three patients were able to undergo liver transplantation (all from a living donor). After they were deemed to have DS, 30 of 43 patients (69.8%) underwent endoscopic interventions, including 29 of 40 (72.5%) who were not able to undergo liver transplantation.

**Fig 3:**
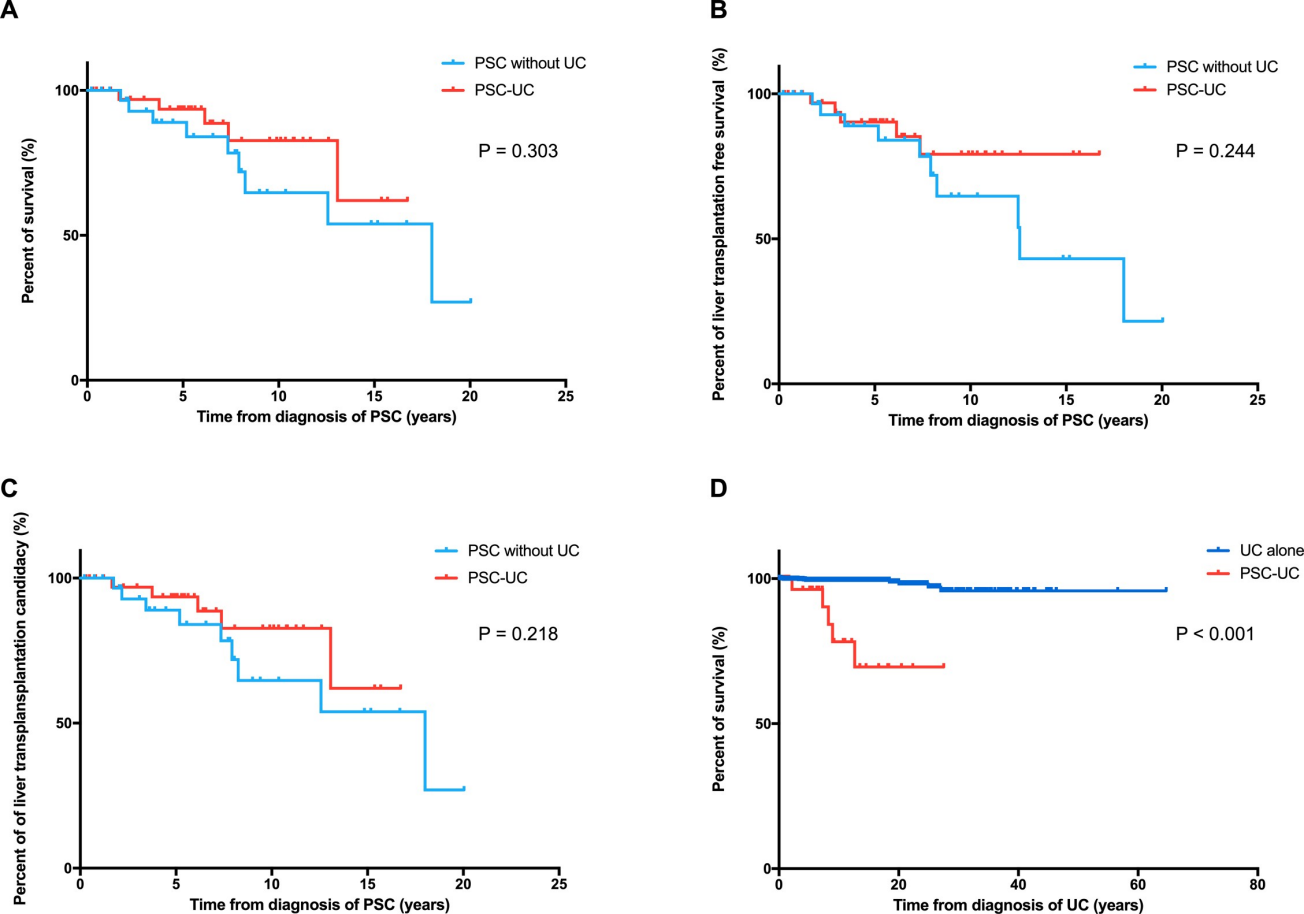
Kaplan-Meier curves of survival (A), liver transplantation free survival (B), time to liver transplantation candidacy (C) from PSC diagnosis, and survival from UC diagnosis (D).

Among 69 PSC patients, 3 (4.4%) had colorectal cancer and 11 (15.9%) had cholangiocarcinoma. During follow-up, 14 patients died (10 with liver dysfunction, 2 with cholangiocarcinoma, 1 with purulent spondylitis, and 1 with multiple myeloma). Median survival and liver transplantation free survival from diagnosis of PSC were 18.0 (95% CI, 11.0–25.0) and 18.0 (95% CI, 10.2–25.8) years, respectively. There were no significant differences in the survival, liver transplantation-free survival, and time to liver transplantation candidacy from PSC diagnosis between the PSC-UC and PSC without UC subjects (Table 1, Fig 3A, 3B and 3C).

### Analysis from the viewpoint of UC population

Table 2 indicate the characteristics and outcomes of UC patients in our cohort. There were 54.6% male patients in all UC cohorts and 54.1% in the UC alone cohort. The PSC-UC cohort had a high ratio of male patients (70.3%) compared with the UC alone cohort (P = 0.052). PSC-UC patients also had a significantly low incidence of left-sided disease (P < 0.001), and high incidence of right-sided disease (P = 0.003) and pancolitis (P < 0.001) compared with those with UC alone. Regarding treatment against UC, proportion of patients who received corticosteroids, immunomodulaters, immunosuppressors, and anti-TNF agents, and patients undergoing colectomy were smaller in the PSC-UC cohort, but not statistically significant, although a trend was observed for both, steroid use and colectomy. In our study, 11 and 774 patients were identified as having a UC flare in the PSC-UC and UC only cohorts, respectively. The cumulative incidence of UC flare (/person-year) was lower in the PSC-US cohorts (P < 0.001).

**Table 2:** Patients’ characteristics and outcomes of UC.

Demographics/characteristics	UC (*n* = 1242)	PSC-UC (*n* = 37)	UC alone (*n* = 1205)	*p*
**Sex**, male, *n* (%)	678 (54.6)	26 (70.3)	652 (54.1)	0.052 ^a^
**Median age at UC diagnosis (range)**	31 (0–87)	27 (4–77)	31 (0–87)	0.392 ^b^
**Extent of UC,** *n* (%)				
Proctitis	218 (17.6)	2 (5.4)	216 (17.9)	0.005 ^a^
Left-sided	277 (22.3)	0 (0.0)	277 (23.0)	< 0.001 ^a^
Right-sided	58 (4.7)	6 (16.2)	52 (4.3)	0.003 ^a^
Pancolitis	500 (40.3)	25 (67.6)	475 (39.4)	< 0.001 ^a^
Undefined/Unknown	189 (15.2)	4 (10.8)	185 (15.4)	0.641^a^
**Medications,** n (%)				
5-ASA	918 (73.9)	26 (70.3)	892 (74.0)	0.608 ^a^
Corticosteroids	618 (49.8)	13 (35.1)	605 (50.2)	0.071 ^a^
Immunomodulator	239 (19.2)	4 (10.8)	235 (19.5)	0.287 ^a^
Immunosuppressor	186 (15.0)	3 (8.1)	183 (15.2)	0.348 ^a^
Anti-TNF agent	147 (11.8)	2 (5.4)	145 (12.1)	0.303 ^a^
**Colectomy,** *n* (%)	152 (12.2)	1 (2.7)	151 (12.6)	0.076^a^
**Colorectal cancer,** *n* (%)	17 (1.4)	2 (5.4)	15 (1.3)	0.089 ^a^
**Cholangiocarcinoma,** *n* (%)	5 (4.0)	4 (10.8)	1 (0.1)	< 0.001 ^a^
**Median survival from diagnosis of UC, years (95% CI)**	Undefined	Undefined	Undefined	< 0.001 ^c^
**5-year survival rate from diagnosis of UC (%)**	99.3	96.2	99.4	
**10-year survival rate from diagnosis of UC (%)**	98.8	76.9	99.4	
**UC flare cumulative incidence/person-years**	0.29	0.06	0.29	< 0.001 ^d^

Abbreviations: PSC, primary sclerosing cholangitis; UC, ulcerative colitis; 95% CI, 95% Confidence interval

^a^ p values were calculated using the Pearson χ2 test or Fisher exact test.

^b^ p values were calculated using the Mann Whitney U test.

^c^ p values were calculated using the log-rank test.

^d^ p values were calculated using the person-years method.

During follow-up, we found two patients (5.4%) with colorectal cancer and four (10.8%) with cholangiocarcinoma in the PSC-UC cohorts. Although there was no statistical significance, PSC-UC patients were more likely to develop colorectal cancer than those with UC without PSC (P = 0.089). Median survival (P < 0.001) from UC diagnosis was significantly shorter in the PSC-UC group than in the UC alone group (Fig 3C). In the present cohort, 10-year survival rates from onset of UC were 76.9% and 99.4% in the patients with PSC-UC and UC alone, respectively.

### Secular changes of incidence in patients with UC and PSC-UC

**Fig 4:**
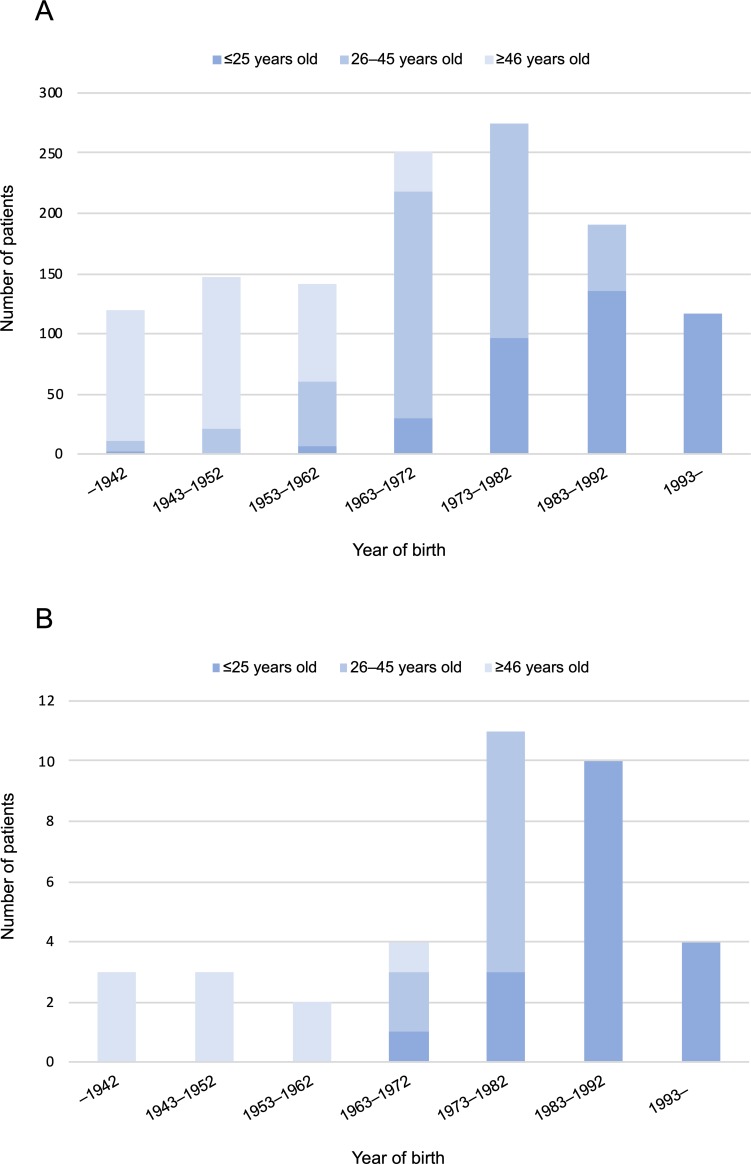
Correlations between date of birth and age at diagnosis in patients with UC (A) and PSC-UC (B).

We confirmed correlations between date of birth and age at onset in patients with UC and PSC-UC (Fig 4). In our study, categories of ages at onset of UC were classified as young (≤25 years), middle age (26–45 years), and old (≥46 years). In Fig 4A, especially young onset (≤25 years) seemed to have increased gradually. This rate might be similar in our PSC-UC patients (Fig 4B).

## Discussion

We characterized clinical features of Japanese patients with PSC-UC from the perspectives of PSC and UC cohorts. As few studies have been done on the clinical characteristics of patients with PSC-UC, there is not enough understanding regarding the ever-changing status of these patients, not only in Japan but also in East Asia [11,35]. Our results warn about the possibility of gradually increasing numbers of patients with PSC-UC in Japan, and it may be similar in East Asian countries, where numbers of patients with UC have increased. We hope that this information helps physicians who are treating either PSC or UC in daily practice, and who believe PSC-UC to be a rare disease in Japan or East Asia.

Comparing our results with those of previous reports, including a Japanese nationwide survey of PSC, we confirmed similar distinctive features of Japanese patients with PSC in that age at onset had two peaks in distribution and younger patients with PSC had a notably high possibility of associated UC [16,17]. Cumulative risk of UC in our patients with PSC was 53.6%, which was higher than what we had expected from former articles. Taken together with correlations between year of birth and age at onset in PSC-UC (Fig 4B), PSC-UC seemed to have increased in the current younger generation, which had become familiarized with Westernized dietary habits. Two population-based cohort studies from Europe (Danish [8] and Sweden [14]) revealed that the incidence of PSC associated with IBD had increased most markedly since 1990, and an increasing PSC incidence was considered to be linked to a continuous increase in UC incidence. Likewise, the incidence of PSC-UC in Japan, especially in the younger generation, might be approaching that of Western countries.

Focusing on the characteristics of UC associated with PSC, we found several unique features concordant with Western patients. First, extent of inflammation in the colon was significantly higher in patients with PSC-UC who more often had right-sided disease (P = 0.003) and pancolitis (P < 0.001) compared with those of patients with UC only in our cohorts [2,36,37]. Second, the degree of UC in patients with PSC-UC was less aggressive, according to the history of medications using corticosteroids (P = 0.071), colectomy (P = 0.076), and occurrence of flares (P < 0.001) [31,32]. Third, overall mortality of patients with PSC-UC was significantly poorer than that of patients with UC (P < 0.001) because prognosis of UC patients is superior to that of PSC-UC patients [8,38]. Fourth, there was a higher probability of colorectal cancer in patients with PSC-UC than in those with UC alone [5,29,38,39]. From East Asia, Ye et al. [35] indicated in an analysis from Korea that patients with PSC-UC had a significantly higher frequency of pancolitis, high incidence of colorectal cancer, and poor prognosis compared with patients with UC only. From Japan, Sano et al. [30] confirmed right side inflammation on histological findings at colonoscopy in patients with PSC-UC, although these data were from small numbers of patients. To the best of our knowledge, our study is the first to compare the characteristics between patients with PSC-UC and UC alone in Japan. These data might suggest that there were no distinct differences in clinical features of PSC-UC between Western and East Asian patients, including Japanese.

Because of an increase in the number of patients with UC in Japan, UC has become a common disease in daily practice and physicians involved in the treatment of UC will increase as well. However, the latest knowledge of risk of PSC in UC patients seems not to be well-known among all Japanese physicians who treat UC, especially those who belong to community hospitals [40]. Our study indicated that the frequency of PSC in Japanese patients with UC was 3.0% and this was not notably different from previous reports worldwide (0.8%–7.6%) [35]. In addition, mild UC patients are treated not only at high-volume centers by specialist in IBDs, but also at local community hospitals by general gastroenterologists, and those mild UC patients seem more frequently to experience combined PSC-UC as mentioned above. The current status of PSC-UC must be shared not only with specialists in UC but also general gastroenterologists in Japan.

Liver transplantation is the only evidence-based effective treatment for PSC and 24% to 50% of patients in Western cohorts undergo liver transplantation [4,7,37]. Due to severe donor shortages of brain death transplantation in Japan, living donor liver transplantation has been the main treatment. Therefore, few patients can undergo liver transplantation compared with other countries. Based on a Japanese nationwide survey of PSC, 54 of 435 patients with PSC (12%) underwent liver transplantation [17]. In our study, though 33.3% of patients were deemed to be liver transplantation candidacies through 16.9 years (median) from onset of PSC, only 4.4% were able to undergo liver transplantation. We also confirmed that most patients with DS did not have a chance to undergo liver transplantation and had undergone endoscopic intervention for palliative dilations of bile duct strictures [28]. Because there is little possibility of a dramatic improvement in the donor shortage in Japan, there are few other ways to find living donor candidacies for liver transplantation in patients with PSC who become liver transplantation candidacies. If patients with PSC, especially those with young onset, gradually increase in the near future, we may consider providing information about living donor liver transplantation to patients with PSC and their family members before becoming candidacies for liver transplantation to increase the choice of donor candidacies.

Although we retrieved a large number of patients from the high-volume center, this study assessed single-center cohorts retrospectively and was not a population-based study or nationwide survey. Thus, we could not evaluate changes in incidence or prevalence rates of patients with PSC and UC. The number of PSC patients was small; therefore, there were insufficient power to detect the differences between the groups, particularly for PSC outcomes. Patients first diagnosed with UC are more likely to have PSC diagnosed at an earlier, milder stage because they are routinely followed up in clinical settings and undergo regular blood tests that may help detect elevated liver enzymes at an early stage of PSC. This lead-time bias can influence PSC outcomes in patients diagnosed with IBD before PSC. Moreover, bias of characteristics or treatments might exist. However, as our study was based on PSC and UC cohorts from a single center, with long observation periods, we could evaluate the clinical courses of patients in more detail.

In conclusion, the numbers of patients with young-onset PSC-UC may be increasing, keeping pace with an increase in UC patients in Japan. The clinical features of Japanese patients with PSC and PSC-UC appeared to be similar to those of Western countries. The current status of PSC, which had changed rapidly, might occur not only in Japan but also in East Asia. The increasing prevalence of PSC-UC in Japan would be a cause of serious clinical issue since the donor shortage of liver transplantation has little possibility of a dramatic improvement. Further large-scale studies including patients with PSC and UC are necessary to clarify current epidemiologic and clinical features more correctly in patients with PSC-UC in Japan and East Asia.

## Supporting information

S1 Table Demonstration data of PSC. (XLSX)

S2 Table Demonstration data of UC. (XLSX)
